# Clinically mild encephalitis/encephalopathy with a reversible splenial lesion associated with *Mycoplasma pneumoniae* infection

**DOI:** 10.1186/s12879-016-1556-5

**Published:** 2016-05-26

**Authors:** Zhe-Feng Yuan, Jue Shen, Shan-Shan Mao, Yong-Lin Yu, Lu Xu, Pei-Fang Jiang, Feng Gao, Zhe-Zhi Xia

**Affiliations:** Department of neurology, The Children’s Hospital of Zhejiang University School of Medicine, 57 Zhugan Xiang, Hangzhou, 310003 Zhejiang Province China

**Keywords:** MERS, Mycoplasma pneumoniae, Corpus callosum, Encephalopathy

## Abstract

**Background:**

Clinically mild encephalitis/encephalopathy with a reversible splenial lesion (MERS) is a clinico-radiological syndrome characterized by transient mild symptoms of encephalopathy and a reversible lesion in the splenium of the corpus callosum on magnetic resonance imaging (MRI). It is often triggered by infection. The common pathogens of MERS are viruses, especially influenza virus. However, *Mycoplasma pneumoniae* (*M.pneumoniae*) are relatively rare pathogens for MERS.

**Case presentation:**

Here we report two paediatric cases of *M.pneumoniae* infection-induced MERS. The diagnosis of *M.pneumoniae* infection was established based on polymerase chain reaction (PCR) and specific serum antibodies (IgM). Both of the two patients presented with mild encephalopathy manifestations and recovered completely within a few days. The initial MRI showed a lesion in the central portion of the splenium of the corpus callosum, which completely resolved on the seventh and eighth day after admission for case 1 and case 2. Lumbar puncture was performed in both patients, which revealed no pleocytosis. In case 1, the patient had hyponatremia, peripheral facial nerve paralysis, and rash. To the best of our knowledge, it is the first MERS case associated with peripheral nerve damage. In case 2, interleukin-6(IL-6) was moderately increased in the cerebrospinal fluid (CSF). It suggested that IL-6 may play a role in the pathogenesis of *M.pneumoniae*-induced MERS.

**Conclusion:**

Our study enriches the available information on the pathogens of MERS and provides valuable data for better understanding of this syndrome.

## Background

Clinically mild encephalitis/encephalopathy with a reversible splenial lesion (MERS) is a clinico-radiological syndrome, which was first reported by Tada et al. in 2004 [1] and further enriched by Takanashi et al [2]. The most common clinical feature is a neurological dysfunction, including delirious behavior, consciousness disturbance, and seizures, all of which recover completely within 1 month [2]. Typical magnetic resonance imaging (MRI) findings of MERS include damage to the splenium of the corpus callosum in the acute phase, and sometimes damage spread to the whole corpus callosum and adjacent white matter. The corpus callosum connects the two cerebral hemispheres, and the splenium is located in the corpus callosum backend. The high-signal lesions are typically symmetrical and notable without enhancement on T2- and diffusion-weighted sequences, especially on the diffusion-weighted sequence. Lesions on the MRI images often disappear in 1 week or a few weeks [1, 2].

MERS may affect adults and children. Its pathogenesis is not clear and often triggered by infection. The most common pathogen is influenza virus A and B, and others include rotavirus, measles virus, adenovirus, *Streptococcus*, and *Escherichia coli* [3]. *Mycoplasma pneumoniae* (*M.pneumoniae*) infection-related MERS is rare [4–6], and MERS has not been previously mentioned in the literature among *M.pneumoniae*-related central nervous system (CNS) dysfunction [7]. Here, we report 2 cases of *M.pneumoniae* infection-induced MERS to enrich the available information on the pathogens of MERS and the pathogenic spectrum of *M.pneumoniae*-related CNS dysfunction.

## Case presentation

### Case 1

A 9-year-old male patient was admitted to the hospital because of fever for 2 days, and headache, vomiting, mouth drooping on the right side, and rash for 1 day. Physical examination showed a body temperature of 38.7 °C and otherwise normal vital signs. Red maculopapular rashes that faded under pressure were scattered all over the body. Neurological examination showed poor mental status and obvious lethargy. Cranial nerve examination revealed mouth drooping on the right side, incomplete eyelid closure and shallow nasolabial sulcus on the left side because of the left peripheral facial paralysis, which is known to be associated with *M.pneumoniae* infection. Meningeal irritation and bilateral Babinski’s signs were negative.

**Fig. 1:**
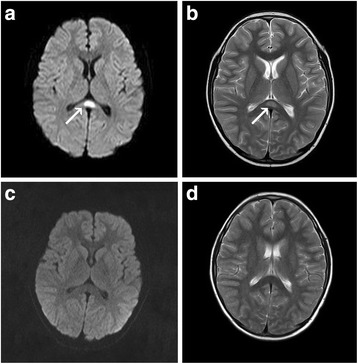
MRI findings of case 1. Diffusion-weighted image (**a**) and T2-weighted image (**b**) on the day of admission showed a focal high-signal lesion in the splenium of the corpus callosum (arrow). On the seventh day after admission, the splenial lesion had completely disappeared on diffusion-weighted image (**c**) and T2-weighted image (**d**)

Peripheral blood analysis showed a leukocyte count of 4500/μL, hemoglobin 12.1 g/dL, platelet count 251,000/μL, and C-reactive protein 16 mg/L(normal range, 0.0–8.0 mg/L). His serum sodium was 127 mmol/L(normal range, 135–145 mmol/L). The pharyngeal swab sample detected *M.pneumoniae* DNA copies 1.1 × 10^4^ per mL by using polymerase chain reaction (PCR). Specific serum *M. pneumoniae* antibodies (IgM) were positive at a titre of 1.71 on the sixth day of the disease (ELISA, normal value:< 1.1). Specific serum IgM antibodies (ELISA) against chlamydia, herpes simplex virus, varicella zoster, cytomegalovirus, Epstein-Barr virus, rubella, toxoplasma, influenza A and B, measles virus, adenovirus, and Japanese encephalitis virus infections were negative. The PCR was negative for influenza A and B in pharyngeal swab. Rotavirus antigen was not detected in his stool specimen. Other blood biochemical tests and urine analysis were normal. The cytokines were not assessed in the serum and cerebrospinal fluid (CSF). CSF was normal with 2 white cell/mL, 20.1 mg/dL protein, and 70.6 mg/dL glucose. *M. pneumoniae* and herpes simplex virus PCR analyses in CSF were negative. The electroencephalogram (EEG) revealed diffuse slow waves. Cranial MRI showed a focal high-signal lesion in the splenium of the corpus callosum on diffusion-weighted and T2-weighted images on the day of admission (Fig. 1a, b).

After admission, treatment included acyclovir (10 mg/kg 8 hourly) for herpes viruses infection before the *M.pneumoniae* infection was confirmed, mannitol (5 ml/kg 8 hourly) for reduction of intracranial pressure, dexamethasone(0.4 mg/kg/d) for reduction of facial nerve swelling, and methycobal (1000 mg/d) for facial nerve paralysis. After *M.pneumoniae* infection was confirmed, azithromycin (10 mg/kg/day) was administered for 5 days. The symptoms improved significantly. Two days after admission, the patient no longer had lethargy, headache, vomiting, and had normal body temperature. Four days after admission, the patient showed improvement in mouth drooping on the right side, incomplete eyelid closure, and stabilization of the rash. Seven days after admission, his serum sodium was 135 mmol/L and his EEG was normal. Re-examination of the cranial MRI, he had normal MRI signals in the corpus callosum (Fig. 1c, d). Ten days after admission, the patient was discharged with excellent mental status, no obvious mouth drooping, substantially restored ability to close the left eyelid, and no rash.

### Case 2

A 12-year-old male patient was admitted to the hospital because of fever for 6 days, and headache, dizziness, vomiting, and cough for 4 days. Physical examination showed a body temperature of 38 °C and otherwise normal vital signs. The breath sound in the right lower lung was low. Neurological examination showed poor mental status and obvious lethargy. Cranial nerve examination did not reveal abnormal findings. Meningeal irritation and bilateral Babinski’s signs were negative.

**Fig. 2:**
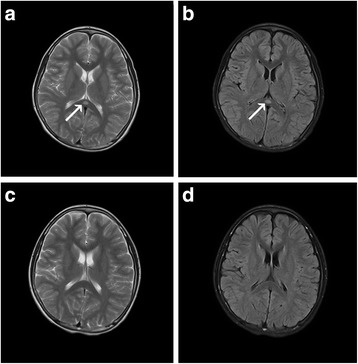
MRI findings of case 2. T2-weighted image (**a**) and FLAIR image (**b**) on the day of admission showed a focal high-signal lesion in the splenium of the corpus callosum (*arrow*). On the eighth day after admission, the splenial lesion had completely disappeared on T2-weighted image (**c**) and FLAIR image (**d**)

Peripheral blood analysis showed a leukocyte count of 8100/μL, hemoglobin 13.0 g/dL, platelet count 253,000/μL, and C-reactive protein 14 mg/L. His serum sodium was 139 mmol/L. The sputum sample detected *M.pneumoniae* DNA copies 1.25 × 10^4^ per mL through the PCR. Specific serum *M. pneumoniae* antibodies (IgM) were positive at a titre of 2.11 on the seventh day of the disease (ELISA, normal value:< 1.1). The above viruses IgM antibodies were also negative. The PCR was negative for influenza A and B in sputum sample and rotavirus antigen was also not detected in his stool specimen. Blood biochemical tests did not show abnormal findings. Cytokines levels were examined in serum and CSF. The results revealed the concentration of interleukin-6(IL-6) was normal in serum and moderately increased in the CSF. The concentration of IL-6 in the serum and CSF was 9 pg/mL and 101 pg/mL(ELISA, normal range < 10 pg/mL), respectively. The levels of other cytokines were normal in serum and CSF. The CSF examination showed no abnormalities. *M. pneumoniae* and herpes simplex virus PCR analyses in CSF were also negative. X-ray suggested right lower lobe pneumonia. The EEG also demonstrated global diffuse slow waves. Cranial MRI showed a focal high-signal lesion in the splenium of the corpus callosum on T2-weighted and fluid-attenuated inversion recovery (FLAIR) images on the day of admission (Fig. 2a, b).

After admission, acyclovir (10 mg/kg 8 hourly) was used for herpes viruses infection. After *M. pneumoniae* infection was confirmed, azithromycin (10 mg/kg/day) was administered for 5 days. Mannitol (5 ml/kg 8 hourly) was used to decrease the intracranial pressure. Two days after admission, the patient showed an improvement in disturbed consciousness and reduced headaches, dizziness, and vomiting. Three days after admission, the patient had normal mental status, normal body temperature, and apparent cough relief. Eight days after admission, the patient was discharged with excellent mental status, no obvious cough, normal EEG findings, and no abnormal MRI signals in the corpus callosum (Fig. 2c, d).

## Discussion

MERS is characterized by acute mild symptoms of encephalopathy occurring during an acute inflammatory disease process. Brain MRIs show characteristic changes in the corpus callosum, and recovery occurs without special treatment. MERS is divided into type 1 (damage limited to the splenium of the corpus callosum on MR images) and type 2 (damage spread to the entire corpus callosum or adjacent white matter or both) [8]. Type 1 MERS is more common.

Most currently reported MERS cases are concentrated in East Asia, especially in Japan, and in children [2, 9]. The most common pathogens are viruses (rotavirus, measles virus, adenovirus, and especially influenza virus) and a few bacteria (Table 1)[3, 9]. However, cases of *M.pneumoniae* infection-induced MERS are relatively rare. *M.pneumoniae* is a common pathogen of respiratory tract infection in children. Therefore, studying pathogens including *M.pneumoniae* can help us understand the etiology and pathogenesis of MERS. CNS disorders such as encephalitis, meningitis, cerebellitis, and myelitis are common extrapulmonary manifestations of *M.pneumoniae* infection, which can also damage the skin and mucosa. However, MERS has not been reported among *M.pneumoniae*-related CNS dysfunction. In the present study, both patients had clinical and imaging manifestations of MERS after *M.pneumoniae* infection, and according to MRI findings, were diagnosed as having type 1 MERS. Notebaert et al. [5] and Zhao et al. [6] each reported 1 case of *M.pneumoniae* infection-induced type 2 MERS, respectively, and Shibuya et al. [4] reported 1 adult case of *M.pneumoniae* infection-induced type 1 MERS. However, little has been reported about child cases of *M.pneumoniae* infection-induced type 1 MERS. In the present study, case 1 had symptoms of not only MERS but also peripheral facial nerve paralysis and rash. To the best of our knowledge, it is the first MERS case associated with peripheral nerve damage, although the peripheral facial paralysis is known to be associated with *M.pneumoniae* infection [10]. This case shows that *M.pneumoniae* infection can cause damage to multiple systems and enriches the spectrum of clinical manifestations of MERS.

**Table 1:** Pathogens of MERS in the literature

Authors	No. of patients	Pathogens of MERS (no. of patients)
Hoshino et al. [9]	153	Influenza (53), rotavirus (18), mumps virus (6), HHV-6 (3), bacterial infection (5)
Takanashi [2]	54	Unknown (22), influenza A/B (6/4), mumps virus (4), adenovirus (3), rotavirus (3), streptococcus (3), Escherichia coli (3)
Tada et al [1]	15	Unknown (10), influenza A (1), adenovirus (1), mumps virus (1), VZV virus (1)
Ka A et al. [11]	7	Unknown (3), influenza B (1), adenovirus (1), CMV virus (1), Salmonella (1)
Bulakbasi et al. [15]	5	Influenza A (5)
Ganapathy et al. [16]	2	Influenza B (2)
Takanashi et al. [14]	4	Kawasaki desease (4)

The pathogenesis of MERS remains unclear. Possible mechanisms include transient intramyelinic edema, interstitial axonal edema, and inflammatory infiltration due to a myelin specific neurotoxin released by the pathogen [1, 2, 11], thus resulting in the transiently reduced diffusion seen on MRI. Takanashi et al. [12] suggested that the process of MERS may involve hyponatremia. Hyponatremia reduces the intracellular osmotic pressure in the splenium of the corpus callosum to facilitate free water entry, leading to transient cerebral edema. In the present study, the early serum sodium level was 127 mmol/L in case 1 and normal in case 2. In addition, the serum sodium level in the case of type 2 MERS reported by Zhao et al. [6] was also within the normal range. It suggests the possible but not essential involvement of hyponatremia in the pathogenesis of *M.pneumoniae*-induced MERS. Some other investigators have suggested the involvement of cytokines. Mori et al. [13] found that the CSF level of IL-6 increased significantly in a child with rotavirus-related MERS, while serum level of interleukin-1β, IL-6, interleukin-8, and tumor necrosis factor-α also increased in MERS children with Kawasaki disease [14], suggesting that the pathogenesis of MERS may involve the activation of the immune system. In the present study, IL-6 was moderately increased in the CSF of patient 2, but was not assessed in the serum and CSF of patient 1. Accordingly, we speculate that IL-6 may be involved in *M.pneumoniae*-induced MERS, but verification of more cases is needed.

MERS, especially type 2 MERS, should be distinguished from other leukoencephalopathies such as acute disseminated encephalomyelitis (ADEM), reversible posterior leukoencephalopathy syndrome (RPLS), and hereditary leukoencephalopathy. In particular, ADEM has clinical manifestations similar to those of MERS during its early stage. However, their MRI findings are different. In ADEM, lesions are distributed asymmetrically and most obvious in the T2-weighted sequence; diffusion is not restricted; enhancement can be observed after use of contrast agents, and the corpus callosum is less likely involved. On the contrary, in MERS, the corpus callosum is often involved symmetrically; the white matter damage is also symmetrical, and diffusion is significantly restricted without enhancement [11]. Through differential diagnosis, MERS can be diagnosed in a timely and precise manner to avoid overtreatment.

## Conclusions

In conclusion, we reported two paediatric cases of type I MERS with coinciding *M. pneumoniae* detection. Our study enriches the available information on the pathogens of MERS and provides valuable data for better understanding of this syndrome. Recognizing this syndrome early in children can avoid overtreatment and provide reassurance about the good prognosis of the disease.

## Abbreviations

ADEM, acute disseminated encephalomyelitis; CNS, central nervous system; CSF, cerebrospinal fluid; EEG, electroencephalogram; IL-6, interleukin-6; MERS, clinically mild encephalitis/encephalopathy with a reversible splenial lesion; MRI, magnetic resonance imaging; PCR, polymerase chain reaction; RPLS, reversible posterior leukoencephalopathy syndrome.
